# The regulatory roles of STAT3 protein in the pathogenesis of viral infections

**DOI:** 10.3389/fcimb.2025.1706270

**Published:** 2025-12-19

**Authors:** Rongrong Gu, Haiyang Zhang, Erying Xu, Shasha Liu

**Affiliations:** 1 Key Laboratory of Animal Pathogen Infection and Immunology of Fujian Province, College of Animal Sciences, Fujian Agriculture and Forestry University, Fuzhou, China; 2 Key Laboratory of Fujian-Taiwan Animal Pathogen Biology, College of Animal Sciences, Fujian Agriculture and Forestry University, Fuzhou, China; 3 Joint Laboratory of Animal Pathogen Prevention and Control of Fujian-Nepal, College of Animal Sciences, Fujian Agriculture and Forestry University, Fuzhou, China

**Keywords:** STAT3 protein, viral infection, interferon, replication, immune response

## Abstract

Signal transducer and activator of transcription 3 (STAT3) serves as a critical regulatory molecule in a multitude of physiological processes, encompassing cell proliferation, differentiation, immune defense, and inflammatory responses. The interaction between STAT3 and viruses is highly intricate, with particular relevance to the viral life cycle. It is widely acknowledged that during viral infection, the phosphorylation of STAT3, which is triggered by upstream activators such as interleukin-6 (IL-6), can exert an effective inhibitory effect on viral invasion. However, accumulating evidence has demonstrated that viruses may exploit their interaction with STAT3 to evade host immune surveillance, thereby facilitating viral persistence. Furthermore, the excessive activation of STAT3 induced by viral infection directly promotes viral replication. The precise mechanism underlying the role of STAT3 in viral infection and pathogenicity remains to be further elucidated. In this review, we summarize recent findings regarding the critical roles of STAT3 in host-virus interactions. Additionally, we discuss the mechanisms of related molecules involved in the antiviral immune response.

## Introduction

The signal transducer and activator of transcription (STAT) family comprises seven structurally related members (STAT1, STAT2, STAT3, STAT4, STAT5α, STAT5β, and STAT6) in mammals, which orchestrate various physiological and pathological processes by participating in JAK-STAT and other related signaling cascades (Coskun et al., 2013; Ma et al., 2022; Xue et al., 2023). Prior to cytokine stimulation, STAT proteins are predominantly localized in the cytoplasm; they mediate cellular responses to cytokines and chemokines, and play a crucial role in the antiviral immune response (Subramaniam et al., 2013).

STAT3 is a multifunctional signaling protein that serves crucial roles in a wide range of physiological and pathological processes, including cell proliferation, differentiation, immune defense, and inflammatory responses (Yu et al., 2009; Durant et al., 2010). In recent years, the association between STAT3 and viral infections has increasingly become a research focus. Notably, STAT3 exhibits dual regulatory roles in the context of viral infections, exerting both pro-viral and anti-viral effects depending on the specific viral species and host micro-environment (Wang et al., 2011; Chang et al., 2018; Hogan et al., 2024). On one hand, it serves as a critical mediator of antiviral immune responses. Specifically, activation of STAT3 is essential for suppressing influenza virus replication (Wang et al., 2019; Liu S. et al., 2023). On the other hand, certain viruses, such as hepatitis B virus (HBV) and hepatitis E virus (HEV), can hijack STAT3-mediated signaling or interact with STAT3 to evade host immune surveillance, thereby facilitating persistent infection (Sooryanarain et al., 2022; Ringelhan et al., 2024). Additionally, viruses like rabies virus (RABV) have been shown to enhance their replicative capacity through the activation of STAT3 signaling pathway (Tu et al., 2019). During the host-virus interaction process, the activity of STAT3 may be either upregulated or downregulated, thereby contributing to viral pathogenesis. Accumulating evidence demonstrates that STAT3 exerts pivotal yet paradoxical roles in the viral replication cycle. In this review, we will systematically discuss the interactions between viruses and STAT3, which are categorized into RNA viruses and DNA viruses.

## The STAT3 protein

### Structural features of STAT3

The genomic localization of STAT3 varies among species. In humans, the STAT3 gene is located on chromosome 17 (17q21.2), spanning from nucleotide position 42, 313, 324 to 42, 388, 568 bp (GRCh38/hg38 assembly). In swines, the STAT3 gene is located on chromosome 12, spanning from nucleotide position 20, 407, 316 to 20, 471, 091 bp. In chickens, the STAT3 gene is located on chromosome 27, spanning from nucleotide position 4, 895, 063 to 4, 910, 486 bp. In mice, the orthologous gene resides on chromosome 11 (11 D; 11 63.82 cM), with genomic coordinates from 100, 775, 924 to 100, 830, 366 bp (GRCm39/mm39 assembly).

**Figure 1:**
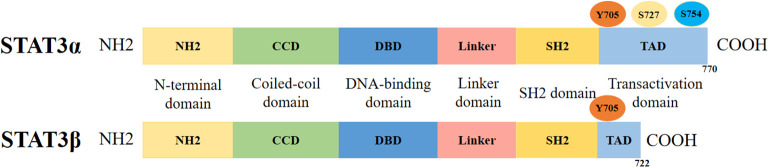
The structure of STAT3 protein. The two splicing variants of the STAT3 protein, STAT3α and STAT3β, are both composed of the following domains: N-terminal domain (ND), the convoluted helical domain (CCD), DNA-binding structural domain (DBD), linker domain (LD), Src homology 2 (SH2) domain, and carboxy-terminal transactivating domain (TAD).

STAT3 protein comprises two splicing variants, STAT3α and STAT3β, each containing six evolutionarily conserved functional domains: the amino-terminal domain (NH2), coiled-coil domain (CCD), DNA-binding domain (DBD), linker domain, SRC homology 2 (SH2) domain, and carboxyl-terminal transactivation domain (TAD) (**Figure 1**). In quiescent cells, the STAT3 protein typically localizes predominantly in an inactive state within the cytoplasm. Upon activation, STAT3 molecules form dimers via reciprocal SH2-phosphotyrosine interactions (Darnell et al., 1994; Pinkham et al., 2016). The SH2 domain represents the most conserved region of STAT3, which plays a pivotal role in signal transduction through binding to specific phosphotyrosine motifs (Sgrignani et al., 2018). Additionally, this domain mediates the formation of STAT3 homodimers, thereby conferring the protein’s transcriptional activity (Hillmer et al., 2016).

### Biological functions of STAT3

First identified in 1994 as a central transcription factor involved in acute-phase inflammatory responses (Roca Suarez et al., 2018), STAT3 has since been demonstrated to regulate a diverse array of biological processes, including inflammation, tissue regeneration, cellular proliferation, differentiation, chemotaxis, and adhesion (Li et al., 2022; Li et al., 2023; Xu et al., 2025). Specifically, the canonical activation pathway of STAT3 involves cytokine-mediated signaling (e.g., interleukin-6 [IL-6] via glycoprotein 130 [gp130]-coupled receptors), wherein ligand-receptor engagement triggers the activation of receptor-associated Janus kinases (JAK1, JAK2, JAK3, and TYK2) (Xu et al., 2024b). Subsequently, STAT3 is recruited to the receptor complex via its SH2 domain, phosphorylated at tyrosine 705 (Y705) by activated JAKs, forms dimers, and translocates to the nucleus to modulate target gene expression (Köhler et al., 2023).

Beyond its canonical transcriptional functions, STAT3 also exhibits phosphorylation-independent activities in cellular respiration, metabolism, autophagy, and viral infection. Specifically, mitochondrially localized STAT3 modulates the function of the electron transport chain, glycolysis, and oxidative phosphorylation (Gough et al., 2009). Emerging evidence further indicates the involvement of STAT3 in autophagic regulation (Shen et al., 2012; Niso-Santano et al., 2013); it participates in processes spanning autophagosome assembly to maturation via both signaling and transcriptional mechanisms. Notably, STAT3 differentially regulates autophagy depending on its subcellular localization: (i) Nuclear STAT3 transcriptionally controls both inhibitors (Bcl-2, BECN1, PIK3C3) and inducers (HIF1A, BNIP3) of autophagy; (ii) Cytoplasmic STAT3 inhibits autophagy through eIF2AK2 and FOXO1/3 suppression; (iii) Mitochondrial STAT3 restricts ROS-triggered autophagy (You et al., 2015). This non-transcriptional functional diversity of STAT3 is closely intertwined with viral infection processes. For instance, the V protein encoded by Mumps virus (MuV) induces the polyubiquitination and proteasome-mediated degradation of STAT3 protein in cells, thereby inhibiting the host’s antiviral immune (Ulane et al., 2003). In contrast, Human cytomegalovirus (HCMV) adopts a distinct strategy by primarily utilizing unphosphorylated STAT3 (uSTAT3)—a key mediator of phosphorylation-independent activities—to promote HCMV DNA replication (Reitsma et al., 2013).

## Characteristics and modes of action of STAT3

STAT3 has emerged as a particularly crucial and extensively studied transcription factor that is widely and ubiquitously expressed across various cell types and tissues, and can be activated by a broad spectrum of cytokines. Upon activation, STAT3 transduces signals from both receptor-associated and non-receptor kinases to the nucleus (Qi and Yang, 2014), where it regulates the expression of downstream target genes, including c-myc, Bcl-xL, Mcl-1, Foxp3, Bcl-2, and CyclinD (Bromberg et al., 1999; Qi and Yang, 2014; De Araujo et al., 2019). Through these target genes, STAT3 participates in governing fundamental cellular processes such as proliferation, survival, differentiation, migration, angiogenesis, inflammation, and autophagy (Aziz et al., 2021; Wu et al., 2021). Under physiological conditions, the activity of STAT3 is tightly regulated by members of the SOCS, PTP, and PIAS protein families (Lee and Cheung, 2019). However, in numerous cancer cell types, STAT3 exhibits constitutive activation and elevated expression levels (Robinson et al., 2019; Parakh et al., 2021; Wang et al., 2022). Notably, uSTAT3 has been increasingly recognized as an important transcriptional regulator in its own right (Yue et al., 2010). Specifically, uSTAT3 can cooperate with transcription factors such as NF-κB to modulate the expression of a distinct set of genes that are typically unaffected by tyrosine-phosphorylated STAT3 (Yang et al., 2007).

## Regulatory functions of STAT3 in RNA viruses infection

**Figure 2:**
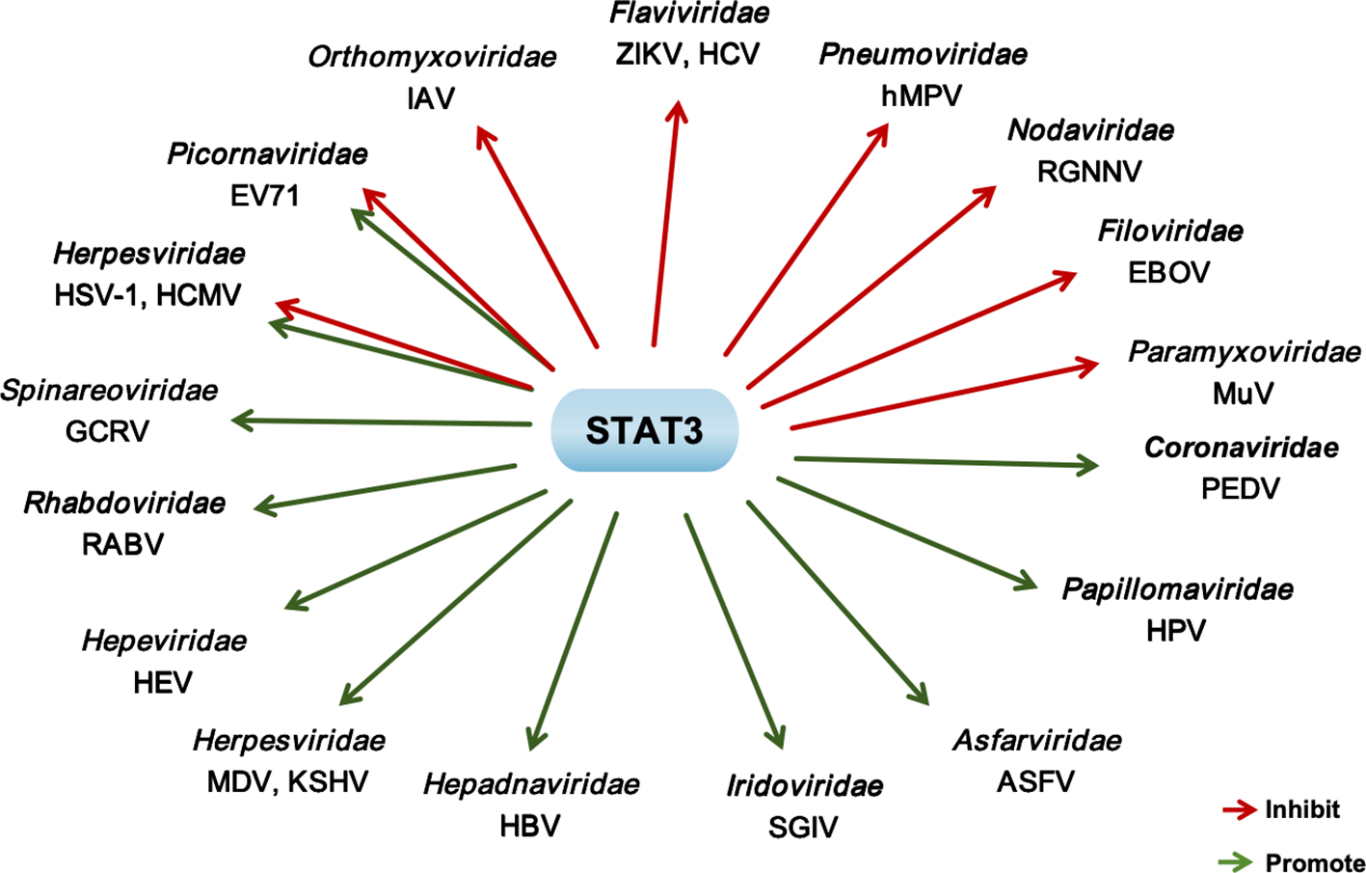
The regulatory effect of STAT3 on the replication of RNA viruses and DNA viruses. Green arrow: Promote; Red arrow: Inhibit.

It is well established that the STAT3 protein can restrict the replication of various RNA viruses. Notably, certain viruses actively suppress STAT3activation to evade its antiviral effects; in the absence of such inhibition, activated STAT3 restricts viral replication by promoting cytokines signaling pathways (Mitzel et al., 2014; Harrison et al., 2021). Conversely, some RNA viruses hijack STAT3 to enhance their proliferation (**Figure 2**). Importantly, the antiviral efficacy of STAT3 is influenced by multiple factors, including viral titer, host cell type, and the phosphorylation status of STAT3, which highlights its context-dependent role during viral infections (**Table 1**). In the following section, we will examine the functions and underlying mechanisms of STAT3 in the context of RNA viruses infection and their contribution to viral pathogenicity.

**Table 1:** The regulatory effect of STAT3 on RNA viruses.

Family	Virus	Functions of STAT3	Mechanism	References
*Flaviviridae*	ZIKV	Inhibit ZIKV infection	Promote the expression of ISGs	(Zhu et al., 2017; Chen et al., 2023)
	HCV	Inhibit HCV infection	Enhance RUNX1-RUNXOR axis through positive feedback mechanism	(Wang and Li, 2018; Thakuri et al., 2020)
*Orthomyxoviridae*	IAV	Inhibit IAV infection	Promote the expression of ISGs	(Liu et al., 2019; Yao et al., 2022; Liu S. et al., 2023)
*Hepeviridae*	HEV	Promote HEV infection	STAT3 inactivation significantly reduces the expression of HEV ORF2 protein	(Sooryanarain et al., 2022)
*Picornaviridae*	EV71	Inhibit EV71 infection	Targeted virus induced miR-124	(Chang et al., 2017)
		Promote EV71 infection	Interference with type I IFN mediated anti-viral response	(Wang et al., 2019)
*Nodaviridae*	RGNNV	Inhibit RGNNV infection	Reduce the transcription levels of autophagy related genes induced by RGNNV infection	(Huang et al., 2015)
*Paramyxoviridae*	MuV	Inhibit MuV infection	STAT3 activates the host’s innate immune response	(Malik et al., 2019)
*Pneumoviridae*	hMPV	Inhibit hMPV infection	/	(Mitzel et al., 2014; Brynes et al., 2024)
*Rhabdoviridae*	RABV	Promote RABV infection	RABV P protein inhibits Gp130 dependent signaling pathway by interacting with STAT3	(Lieu et al., 2013; Tu et al., 2019)
*Filoviridae*	EBOV	Inhibit EBOV infection	VP24 inhibits the STAT3 mediated IL-6 cytokine signaling pathway, relieving the inhibitory effect of STAT3 on virus replication	(Harrison et al., 2021)
*Phenuiviridae*	RVFV	/	Reduce cell death	(Pinkham et al., 2016)
*Spinareoviridae*	GCRV	Promote GCRV infection	Assisting virus invasion and inhibiting host anti-viral immunity	(Hou et al., 2023; Hu et al., 2024; Yang et al., 2024)
*Coronaviridae*	PEDV	Promote PEDV infection	Negative regulation of type I IFN response weakens host anti-viral ability	(Yang et al., 2018)

“/” represent current research gaps.

### Flaviviridae

STAT3 can efficiently restrict the replication of Zika virus (ZIKV) and hepatitis C virus (HCV). In the context of ZIKV infection, the infection of primary retinal glial cell induces the phosphorylation of STAT3 at Tyr705 (Zhu et al., 2017). Moreover, splicing factor 3b subunit 3 (SF3B3) interacts with ZIKV non-structural protein 5 (NS5); notably, the overexpression of SF3B3 in U251 cells further enhances STAT3 phosphorylation, which in turn promotes the expression of IFN-stimulated genes (ISGs) to inhibit ZIKV replication (Chen et al., 2023). During HCV infection, STAT3 is involved in complex regulatory networks that suppresses viral replication and immune responses. Runt-related transcription factor 1 (RUNX1) and its overlapping RNA (RUNXOR) (Haridy et al., 2023) form a mutual positive regulatory loop to modulate the STAT3-miR-124 axis, thereby controlling the expression of immunosuppressive molecules in myeloid-derived suppressor cells (MDSCs). Here, STAT3 reinforces the RUNX1-RUNXOR axis through a positive feedback mechanism and negatively regulates miR-124 expression, and this synergistic effect promotes MDSC expansion while inhibiting HCV replication during chronic infection (Thakuri et al., 2020). In addition, miR-29c expression is downregulated in human hepatocellular carcinoma (Huh7) cells infected with HCV JFH-1 strain, accompanied by upregulated STAT3 mRNA and protein levels. Mechanistically, STAT3 is a direct target of miR-29c, and miR-29c inhibits HCV infection by promoting type I IFN responses through targeting STAT3 expression in JFH-1-infected Huh7 cells (Wang and Li, 2018).

### Orthomyxoviridae

Influenza A virus (IAV) (Liu H. et al., 2023), belonging to *Orthomyxoviridae* family, has aroused a global health concern (Wang et al., 2016; Chen et al., 2024; Chi et al., 2024). Disruption of STAT3 by shRNA-mediated interference enhances IAV replication, whereas STAT3 overexpression reduces viral replication. Notably, studies have demonstrated that at the early stage of IAV infection—prior to the production of cytokines such as IL-6—phosphorylation of STAT3 at Tyr705 is induced in A549 cells. This STAT3 phosphorylation event is dependent on RIG-I/MAVS/Syk axis and plays a critical role in suppressing IAV replication. In terms of mechanism, STAT3 exerts antiviral functions by inhibiting the excessive production of type I and III IFNs; specifically, studies have shown that STAT3^Y705F/+^ mice with knockout of type I or type III IFN receptors exhibit significantly enhanced resistance to IAV infection compared with STAT3^Y705F/+^ mice (Liu S. et al., 2023). Conversely, to fulfill its replication requirements, IAV has evolved strategies to evade STAT3-mediated suppression. Specifically, one study has clarified that IAV has circumvents IL-6/STAT3-dependent antiviral innate immune responses by upregulating the production of SOCS3 (Liu et al., 2019). Moreover, high-dose pH1N1 infection induces an excessive early immune response, characterized by massive neutrophil recruitment, cytokines storm, and STAT3 activation. In contrast, STAT3 exerts an immuno-protective effect during low-dose pH1N1 infection (Yao et al., 2022), highlighting the dose-dependent duality of STAT3’s role in IAV pathogenesis.

### Hepeviridae, picornaviridae, and nodaviridae

Beyond the aforementioned viruses, STAT3 can also effectively influence the infection and pathogenic processes of other single-stranded positive-sense RNA viruses, including HEV, enterovirus 71 (EV71), and grouper nervous necrosis virus (RGNNV), which belongs to *Hepeviridae (*Wang and Meng, 2021), *Picornaviridae (*Tang et al., 2014), *Nodaviridae (*Vázquez-Salgado et al., 2024), respectively. Inactivation of STAT3 significantly reduces the expression of HEV ORF2 protein, indicating that STAT3 promotes HEV replication (Sooryanarain et al., 2022). However, the role of STAT3 in EV71 replication is highly dependent on the host cell type. In human rhabdomyosarcoma (RD) cells, shRNA-mediated STAT3 interference enhances EV71 replication, whereas STAT3 overexpression inhibits viral replication, demonstrating that STAT3 suppresses EV71 replication in RD cells. Mechanistically, the antiviral activity of STAT3 in RD cells is partially antagonized by EV71-induced miR-124, which directly targets the 3’ untranslated region (UTR) of STAT3 mRNA to downregulate its expression (Chang et al., 2017). In contrast, in EV71-infected glial cells, phosphorylation of STAT3 at Tyr705 or Ser727 sites induces the increased expression of immunomodulatory factors, thereby promoting EV71 replication (Wang et al., 2019). In RGNNV-infected fish cells, the subcellular distribution of phosphorylated STAT3 (Ec-STAT3) is altered, and the activity of the STAT3 promoter is significantly upregulated, indicating that STAT3 activation is involved in RGNNV infection. Notably, suppression of Ec-STAT3 markedly increases the transcriptional levels of autophagy-related genes induced by RGNNV infection, suggesting that Ec-STAT3 inhibits viral replication in these cells (Huang et al., 2015).

### Paramyxoviridae, pneumoviridae, and phenuiviridae

STAT3 also modulates the infection process and regulates cell death in the context of infections by MuV, human metapneumovirus (hMPV), and Rift Valley fever virus (RVFV). For MuV, studies have found that the viral V protein (MuV V) forms a ubiquitin E3 ligase complex by recruiting cellular components, including DDB1 and Cullin4A, to specifically target the STAT3 protein for proteasome-mediated degradation (Ulane et al., 2003; Puri et al., 2009). Notably, a single amino acid mutation (E95D) in MuV V alters the protein’s ability to induce STAT3 degradation. Functional validation shows that inhibition virus-mediated STAT3 degradation accelerates viral clearance, directly confirming that STAT3 exerts antiviral effects during MuV infection (Malik et al., 2019). In the case of hMPV, either viral infection or transfection with the hMPV small hydrophobic (SH) protein blocks IL-6-mediated STAT3 activation, thereby promoting hMPV replication (Brynes et al., 2024). Mechanistically, hMPV employs multiple strategies to disrupt STAT3 function and facilitate its own propagation, including suppressing STAT3 nuclear translocation, impairing its transcriptional activity, and inducing STAT3 protein degradation (Mitzel et al., 2014). Regarding RVFV, studies indicate that RVFV infection induces phosphorylation of STAT3 at Tyr705 in Vero cells, human small airway epithelial cells (HSAECs), and mouse embryonic fibroblasts (MEFs)—a process primarily dependent on the viral non-structural (NS) protein. Intriguingly, STAT3 deletion has little effect on RVFV replication but renders cells more susceptible to RVFV-induced cell death, suggesting that STAT3 plays a cyto-protective role during RVFV infection by mitigating virus-induced cytotoxicity, independent of its direct antiviral activity (Pinkham et al., 2016).

### Rhabdoviridae and filoviridae

Two other important zoonotic pathogens—RABV, *Rhabdoviridae* family (Lian et al., 2022) and Ebola virus (EBOV), *Filoviridae* family (Shu et al., 2019)—also exhibit distinct interactions with STAT3. In the context of RABV infection, conflicting yet context-dependent regulatory patterns have been observed. Specifically, RABV infection induces excessive activation of STAT3 in baby hamster kidney fibroblasts (BHK-21), which in turn inhibits viral replication (Tu et al., 2019). In contrast, another study reveals that the RABV P protein enhances viral replication by mediating the inhibition of glycoprotein 130 (Gp130)-dependent signaling via interaction with STAT3 (Lieu et al., 2013). This dual regulatory effect suggests that the role of STAT3 in RABV infection is highly dependent on cellular context and viral protein interactions. EBOV, by contrast, adopts a distinct strategy to suppress STAT3 function for immune escape. Following infection, the EBOV VP24 protein antagonizes STAT3 to block IL-6-mediated signaling—consistent with IL-6 being one of the key cytokines that activate STAT3 during infections with various viruses (e.g., SARS-CoV-2, respiratory syncytial virus, and IAV) (Morgan and Macdonald, 2019; Patra et al., 2020). Mechanistic analyses indicate that VP24 employs dual mechanisms to antagonize STAT3-containing dimers: it inhibits STAT3-STAT1 heterodimers via a karyopherin-dependent pathway, while suppressing STAT3 homodimers through a karyopherin-independent mechanism (Harrison et al., 2021). This targeted disruption of STAT3 signaling enables EBOV to evade host antiviral defenses and promote its own replication.

### Spinareoviridae and coronaviridae

Grass Carp Reovirus (GCRV, *Spinareoviridae* family) (He et al., 2024) and Porcine Epidemic Diarrhea Virus (PEDV, *Coronaviridae* family) (Chen et al., 2015) have also been shown to promote their own replication by manipulating the STAT3 signaling pathway. For GCRV, it utilizes temperature-dependent activation of IL-6/STAT3 signal transduction to promote viral entry and replication. This “temperature switch” is validated through a temperature-switch experiment: *Ctenopharyngodon idellus* kidney (CIK) cells are first infected with GCRV at 18°C or 28°C for 8 hours, followed by switching the incubation temperature to 28°C or 18°C, respectively. Results show that the temperature switch increases STAT3 protein levels, enhances STAT3 phosphorylation, and simultaneously induces the production of IL-6 and heat-shock-related chaperone protein HSP90. Mechanistically, HSP90 facilitates the interaction between viral proteins and host cell receptors, thereby enhancing GCRV entry and replication (Hou et al., 2023). Consistent with this, Illumina RNA sequencing data show that the levels of STAT3 are significantly upregulated in GCRV-infected grass carp compared with the uninfected control group (Yang et al., 2024). Notably, GCRV has also evolved an immune evasion mechanism that downregulates IFNs expression by hijacking the IL-6/STAT3 axis. Specifically, compared with infection at 18°C, GCRV infection at 28°C significantly induces IL-6 expression and STAT3 phosphorylation. The activated STAT3 then inhibited IRF3 nuclear translocation via interacting with it, thereby downregulating IFNs expression and creating an immunosuppressive environment conducive to viral replication (Hu et al., 2024).

Regarding PEDV, accumulating evidence clarifies its proviral regulation of the STAT3 signaling pathway through a well-defined molecular mechanism. PEDV infection induced both STAT3 phosphorylation and epidermal growth factor receptor (EGFR) activation. Functional validation shows that treatment with the STAT3-specific inhibitor S3I-201 significantly increases IFN-β mRNA levels in PEDV-infected porcine small intestinal epithelial (IPEC-J2) cells and human embryonic kidney 293 (HEK293) cells. Mechanistically, EGFR activation impairs the antiviral activity of type I IFN, a process that requires the involvement of STAT3—an downstream effector of the EGFR signaling cascade. This EGFR-STAT3 axis forms a regulatory loop that suppresses host antiviral immunity, thereby facilitating PEDV replication (Yang et al., 2018) (**Figure 2**). Importantly, this finding aligns with emerging evidence that STAT3 negatively modulates type I IFN-mediated responses across diverse viral infection models (Ho and Ivashkiv, 2006), underscoring the evolutionary conservation of STAT3 hijacking as a viral immune escape strategy.

## Regulatory roles of STAT3 during DNA viruses infection

Recent studies have progressively unraveled the complex interplay between STAT3 and DNA viruses. Consistent with its regulatory role in RNA virus infections, STAT3 exerts a dual effect on DNA viruses, capable of either inhibiting or promoting viral replication. Notably, even under the same viral infection context, STAT3 may switch between pro-viral and anti-viral functions depending on the specific physiological conditions of the host. This functional versatility and context dependence underscore the necessity of conducting further in-depth investigations into the roles of STAT3 in DNA virus infections and their underlying pathogenic mechanisms (**Table 2** and **Figure 2**).

**Table 2:** The regulatory roles of STAT3 in DNA viruses.

Family	Virus	Functions of STAT3	Mechanism	References
Herpesviridae	HSV-1	Inhibit HSV-1 infection	Inducing the production of anti-viral molecules such as IFN-α and ISGs	(Lee and Cheung, 2019)
		Promote HSV-1 infection	Promote the expression of NEAT1 and provide support for viral gene transcription	(Wang et al., 2017)
	MDV	Promote MDV infection	Inhibition of ATR-Chk1 pathway	(Lian et al., 2019)
	HCMV	Inhibit HCMV infection	Regulating downstream gene expression such as SOCS3 and regulating nuclear localization	(Reitsma et al., 2013)
		Promote HCMV infection	Promoting cell proliferation indirectly promotes HCMV replication	(Slinger et al., 2010)
	KSHV	Promote KSHV infection	Support virus induced cell proliferation and transformation, indirectly promoting virus replication	(Gruffaz et al., 2017)
Hepadnaviridae	HBV	Promote HBV infection	Activation of Tyr705 site	(Yang et al., 2016; Ringelhan et al., 2024)
Iridoviridae	SGIV	Promote SGIV infection	Up-regulating the expression of pro-survival genes	(Huang et al., 2014)
Asfarviridae	ASFV	Promote ASFV infection	Inhibition of host cell apoptosis through the CD2v-CSF2RA-JAK2 pathway	(Gao et al., 2023)
Papillomaviridae	HPV	Promote HPV infection	By forming positive feedback with HPV oncogenes, maintaining the viral oncogenic phenotype indirectly supports persistent HPV related infections	(Morgan and Macdonald, 2019; Strobel et al., 2023)

### Herpesviridae

Several viruses of the *Herpesviridae* family, including HSV-1, MDV, HCMV, and KSHV have aroused global health concern (Widener and Whitley, 2014; Broussard and Damania, 2020; Shi et al., 2020; Yu et al., 2021). And studies have demonstrated that they exhibit diverse interactions with STAT3. HSV-1 shows a bidirectional relationship with STAT3. On one hand, STAT3 has an anti-HSV-1 effect. The STAT3 knockout (KO) mice are more susceptible to HSV-1 infection than wild-type mice, characterized by higher viral loads and more significant weight loss. Bone marrow-derived macrophages (BMMs) from STAT3 KO mice exhibit reduced levels of IFN-α and ISGs after HSV-1 infection (Lee and Cheung, 2019). On the other hand, STAT3 can promote the replication of HSV-1. Wang et al., find that the long non-coding RNA (lncRNA) NEAT1 significantly increases 2 h after HSV-1 infection and reach its peak at 4 h in human cervical cancer (HeLa) cells. However, silencing STAT3 expression with STAT3 siRNA significantly decreased NEAT1 expression and suppressed HSV-1 replication. Mechanistically, HSV-1 infection promoted the expression of NEAT1 in a STAT3-dependent manner, facilitates viral gene transcription, and ultimately enhances viral replication (Wang et al., 2017). However, MDV only interacts with STAT3 to favor its own replication. Infection with MDV leads to a gradual increase in the levels of STAT3 protein and STAT3 phosphorylation in chicken embryonic fibroblasts (CEF), eventually inhibiting the ATR-Chk1 pathway and facilitating viral replication (Lian et al., 2019).

HCMV also has complex interactions with STAT3. The HCMV IE1 protein disrupts IL-6-induced STAT3 phosphorylation and impairs the binding of STAT3 to the SOCS3 promoter. Finally, nuclear localization or expression of STAT3 is suppressed, while viral gene expression is disrupted, and the viral amplification of HCMV is decreased (Reitsma et al., 2013). However, the HCMV-encoded G protein-coupled receptor US28 establishes a positive feedback pathway through IL-6-dependent activation of JAK1-STAT3 axis, promoting the proliferation of infected cells and adjacent cells (Slinger et al., 2010). In addition, KSHV facilitates its replication through the activation of STAT3 as well. In KSHV-transformed cells, the chronic induction of IL-6 mediates the constitutive activation of STAT3 pathway, which is a critical contributor to uncontrolled cell proliferation and transformation (Gruffaz et al., 2017).

### Hepadnaviridae, iridoviridae, asfarviridae, and papillomaviridae

Studies have demonstrated that HBV, SGIV, ASFV, and HPV interact with STAT3 to facilitate their infection processes. HBV infection is one of the major causes of HCC development. HBV-induced mitochondrial ROS overproduction enhances the activation of STAT3 and hepatocellular carcinoma (HCC) development. Mechanistically, ROS-mediated DNA methylation repressed SOCS3 expression through hypermethylation of the SOCS3 promoter, contributing to the sustained activation of IL-6/STAT3 (Yuan et al., 2016). Moreover, the development of HBV infection-related HCC in mice is STAT3-dependent. After hybridizing HBV-infected mice with hepatocyte-specific STAT3 conditional knockout mice, the development of HCC abolish (Ringelhan et al., 2024). STAT3-shRNAs promote HBV positive HCC cell apoptosis, and suppresses HBV replication, which broke HBV-STAT3 signaling loop and augment STAT3-shRNAs-mediated anti-HCC effect (Yang et al., 2016). Similarly, STAT3 phosphorylation in grouper splenocytes could be induced during SGIV infection. Importantly, inhibition of STAT3 activation significantly reduces SGIV replication in cells (Huang et al., 2014). Together, HBV and SGIV rely on the activation of STAT3 to support their replication cycles.

CD2v, the major envelope glycoprotein of ASFV (Hong et al., 2022; Lu et al., 2023; Xu et al., 2024a), enhances STAT3 phosphorylation and nuclear translocation. Deletion of CD2v downregulates the JAK2-STAT3 pathway and inhibits ASFV replication, directly demonstrating that the JAK2-STAT3 pathway facilitates ASFV replication (Gao et al., 2023). Consistent with this, targeted inhibition of STAT3 phosphorylation using specific small-molecule inhibitors also suppresses ASFV replication, further confirming STAT3’s proviral role in ASFV infection (Geng et al., 2025). Similarly, the interaction between HPV and STAT3 contributes to both viral replication and carcinogenesis. STAT3 interacts with HPV oncogenes E6/E7 to enhance their expression; conversely, the expression of HPV oncogenes E5, E6, and promotes STAT3 activation, forming a positive regulatory loop that sustains viral persistence and cellular transformation (Strobel et al., 2023). Additionally, IL-6 secreted by HPV-positive cervical cancer cells induces STAT3 activation in neighboring HPV-negative cervical cancer cells, thereby enhancing cancer cell proliferation (Morgan and Macdonald, 2019) (**Figure 2**). This paracrine regulation mediated by the IL-6/STAT3 axis highlights the multifaceted role of STAT3 in HPV-associated tumorigenesis beyond direct viral regulation.

## Conclusions

The interaction between viruses and their host organisms is extremely complex. A diverse range of viruses—IAV, GCRV, HBV, HPV, and KSHV—can directly or indirectly induce STAT3 phosphorylation. This is accomplished either by enhancing the production of upstream STAT3 agonists (e.g., IL-6) or inhibiting its negative regulatory factors (e.g.,SOCS3). In contrast, to establish an intracellular environment conducive to their own replication and persistent infection, viruses have evolved specialized mechanisms to counteract host antiviral defenses. For instance, viruses such as MuV, HCMV, EBOV, and hMPV can disrupt the STAT3 signaling pathway through multiple strategies, including inducing STAT3 degradation, blocking its phosphorylation, promoting its excessive activation, interfering with its nuclear translocation, or reducing its transcriptional activity. Additionally, viruses such as EV71 and HSV-1 exhibit functional divergence across different host cell types, where their pro-viral or anti-viral effects are largely determined by the specific phenotypic and functional characteristics of the host cells.

In-depth investigation of the interaction between viruses and host STAT3 protein holds profound significance for further elucidating the host anti-viral defense mechanisms and the virus immune escape strategies. Although considerable progress has been achieved in this field in recent years, the intrinsic regulatory networks underlying STAT3-mediated anti-viral immune responses have not yet been fully unraveled. Additionally, the specific pro-viral or anti-viral mechanisms exhibited by certain viruses during infection remain incompletely characterized. A more comprehensive elucidation of these mechanisms is expected to deepen our understanding of the role of STAT3-related regulatory network in anti-viral immunity, and further provide novel insights for the development of therapeutic strategies targeting STAT3.

## Glossary

ASFV: African swine fever virus
BMMs: Bone marrow-derived macrophages
EBOV: Ebola virus
EV71: Enterovirus 71
EGFR: Epidermal growth factor receptor
Gp130: Glycoprotein 130
GCRV: Grass Carp Reovirus
HBV: Hepatitis B virus
HCC: Hepatocellular carcinoma
HCV: Hepatitis C virus
HCMV: Human cytomegalovirus
HEV: Hepatitis E virus
hMPV: Human metapneumovirus
HPV: Human papilloma virus
HSV-1: Herpes simplex virus-1
HSP90: Heat Shock Protein 90
IAV: Influenza A virus
IFN: Interferon
IL-6: Interleukin-6
lncRNA: Long non-coding RNA
ISGs: IFN-stimulated genes
IRF3: Interferon Regulatory Factor 3
KSHV: Kaposi’s Sarcoma-Associated Herpesvirus
KO: Knockout
MDSCs: Myeloid-derived suppressor cells
MDV: Marek’s disease virus
MuV: Mumps virus V
NS5: Non-structural protein 5
PEDV: Porcine Epidemic Diarrhea Virus
RABV: Rabies virus
RGNNV: Grouper nervous necrosis virus
ROS: Reactive Oxygen Species
RVFV: Rift Valley fever virus
SF3B3: Splicing factor 3b subunit 3
SOCS3: Suppressor of cytokine signaling 3
STAT3: Signal transducer and activator of transcription 3
SGIV: Singapore grouper iridovirus
ZIKV: Zika virus
